# Treatment of Puerperal Fever

**Published:** 1851-03

**Authors:** E. Murphy


*Treatment of Puerperal Fever.* By E. Murphy, M. D. —[As a general rule we avoid the republication of the routine lectures which are now so commonly committed to the pages of the weekly journals, but puerperal fever is a disease of such moment, and it is so important to the practitioner to have the most enlightened views on the subject of treatment at his "fingers' ends," as it were, that we offer no apology for departing from our usual custom. After an admirable history of the various types of child-bed fever, their symptoms and pathology, Dr. Murphy thus proceeds:]

The treatment of puerperal fever must be considered in reference to the views of the malady above stated. It divides itself into two questions: First, the prophylactic treatment. In discussing this question, I must ask the reader to assume with me that it is contagious. If it be not, precaution taken can do no harm. On the other hand, if contagious, the neglect of such means of prevention would do infinite mischief. We have already alluded to the rapid diminution and ultimate disappearance of puerperal fever in the Dublin Lying-in Hospital, when Dr. Collins put into force active sanatory measures. The chief agent employed by him was chlorine. Dr. Routh gives an interesting account of the means adopted by Dr. Semelweiss, for preventing the spread of the disease. "He recommended all students frequenting the division (the labor-wards) not to handle dead matter, or if they did, he forbade them to make any examination till the following day. In the second place, he directed all the students who attended the practice of the division *to wash their hands in a solution of chlorine prior to and after every (vaginal) examination made on the living subject.* The result of these precautionary measures was that the number of deaths at once fell to seven per month." They had been from 30 to 40 per month, and we can assign no other cause for this great difference of mortality than the means used by Dr. Semelweiss for preventing the communication of the disease. The prophylactic was chlorine, and hence, comparing the facts stated by Dr. Collins with those of Dr. Semelweiss, this gas seems to have the power of destroying the poison. In order, therefore, to prevent the diffusion of puerperal fever, chlorine is essential. Solutions of it should be freely used, the hands washed, and the sheets of the bed sprinkled with it, even at the risk of damaging the texture. The vapor of chlorine should also be diffused through the apartment. I am inclined to think that the chlorates of soda or potass taken internally would be useful on the same principle. Besides these means the practitioner should also be careful, if in the attendance of a case of puerperal fever, to change his dress before he visits another patient in labor. The fomites of the fever have been known to lurk about the dress of the attendant, and produce it after a period much later than might be imagined. In one ward of the Dublin Lying-in Hospital the fomites remained for months, and renewed the disorder. If proper precautions are used, I am satisfied that there is no danger of communicating puerperal fever; but I fear in some instances a want of caution has had this effect. The practitioner may have been led astray by the theorist, who tells him that puerperal fever is nothing but a local inflammation, which we know is not contagious; or he may imagine there is no risk because some of his patients may have escaped contamination. Errors of this kind, we have reason to think, have led to the dissemination of poison, and should be carefully guarded against.

*The remedial treatment* has been a warm subject of controversy, chiefly arising from the different views of the disease that have been advocated. Those who hold the doctrine of local phlegmasia defend an active antiphlogistic treatment; while the advocates of the epizootic character of puerperal fever maintain the importance of supporting the constitution against the attack. It appears to us that the treatment of both, to a certain extent, is correct, although the principle on which the antiphlogistic plan is founded we believe to be erroneous. Depletion, purgatives, emetics, certainly reduce inflammation, because they diminish the quantity of the blood and the velocity with which it is impelled by the heart; but the very same means, by lessening the quantity of poisoned blood, and by acting as evacuants to discharge a poison from the circulation only, renders the blood more pure and less dangerous than it was before. These remedies are beneficial, therefore, not as antiphlogistics, but as antiseptics. If we act on such a principle the only question we have to decide is—can the patient bear the treatment? Has the constitution sufficient power to recover itself from the loss occasioned by the treatment when it is already prostrated by the influence of a poison? We believe that this question will admit of no general answer; we can only speak with a special reference to the constitution and the conditions in which it is placed. If the dose of poison be excessive, evacuants are inadmissable, because the constition has no power, no action. On this principle we object to depletion in Armstrong's "congestive disease," and consider Mackintosh's doctrine of "latent peritonitis" altogether fallacious when applied to puerperal fever. If but little be absorbed, and the prominent symptoms are of a sthenic character, evacuants are the most efficient, because the poison is rapidly removed, any local inflammation is promptly subdued, and the constitution soon recovers itself. Between these extremes there is every shade of intensity in the disease, which almost renders each case a separate subject of consideration. In some the evacuant treatment, in others the stimulant, is successful. In those situations where the poison is concentrated, as in hospitals, or in places where it suddenly appears and commits great devastation, we think that the stimulant practice is most advisable. The remarkable success of Dr. Copland's practice in the Queen Charlotte's Hospital is a sufficient illustration of this truth, and may be contrasted with the failure of Armstrong's treatment in the Dublin Lying-in Hospital, about the same period. On the other hand, when the poison is diffused, as in private practice, evacuants may be employed with advantage; at the same time, it is right to observe that the natural strength of the patient's constitution forms an important element in determining this question. It is very well known that the strength of constitution varies with the locality. The inhabitants of London, for instance, do not bear the same activity of antiphlogistic treatment as those of Dublin or Edinburgh. The residents of a country district, breathing a purer air, and enjoying active exercise, or occupied in rustic duties, have stronger constitutions than citizens; and thus, when we would wish to form a fair estimate of any proposed treatment by the evidence of its success which is adduced, it is very important to inquire where the cases treated have lived. Dr. Gordon's and Mr. Hey's were chiefly rural practices. Dr. Armstrong's and Dr. Mackintosh's were scattered over a town; and although each met with cases of intense puerperal fever, they were exceptions, not the rule; we have every reason to think that their "clientelle" consisted of patients with naturally strong constitutions that would admit of free evacuation. You may meet, however, and in cities often will meet, with patients whom you cannot bleed, and who require stimulants; we cannot, therefore, lay down a general rule, unless it be that of Sydenham—"to study the constitution of the year," and we would add, "of the place." Having premised these general observations, we shall proceed to consider separately the remedies usually employed; and first, with regard to the evacuants.

*Depletion* is more generally employed than any other mode of treament. Dr. Ferguson, after a most careful and cautious inquiry, gives the following as the sum of his experience of bleeding as a remedy in puerperal fever:

"Of all the means we possess of arresting this malady, I believe bleeding, general or local, to be far the most extensively applicable. The cases in which it is not so are exceptions to the rule." We fully agree in this opinion, but we do not consider depletion efficacious because it is antiphlogistic, but because it is a most rapid evacuant. On this principle alone can we explain the efficacy of the very bold depletion sometimes employed. We do not treat peritonitis by taking away large quantities of blood; and there are some forms of it in which we cannot bleed at all. Yet in puerperal fever Hey took twenty, thirty, even fifty ounces of blood from the arm. Mackintosh says—"In some fortunate cases, the histories of which were lost in my travels, I can well remember abstracting forty and fifty ounces at one bleeding." In order that depletion be efficient it should be promptly undertaken the the moment that a rigor gives evidence that the poison is absorbed; the removal of blood then is attended with the best effects; but at a later period, when time is given to alter its properties, and to soften the tissues, when effusions take place, depletion has a contrary effect; the strength of the patient at once gives way, and death rapidly follows. Such we believe to be the principle on which depletion, when it may be adopted, is useful; but you must always bear in mind that it may be quite inadmissible; you must carefully inquire into the character of the epidemic, the constitution of your patient, and, as I have said, the constitution of the place. If you seek among the symptoms for a guide to determine your practice, I know of none we can depend on but the pulse, and even that is uncertain. If the pulse be firm and wiry you may bleed, but if it be compressible and unsteady you cannot do so. In the former case the constitution is struggling against the disease, and manifests a degree of vigor that will admit of depletion. In the latter, it is rapidly giving way under the attack, and depletion only hastens debility and its consequence, death; but although this is generally true there often exceptions in both cases which may deceive you. Depletion, therefore, sometimes must be tried as an experiment, and, if so, should be had recourse to as soon after the rigor as possible; at a later period it should never be employed. In hospital practice, and with women in the lower walks of life, who have been subjected to privations or causes of mental depression, we would not think depletion advisaable.

*Purgatives*, also, act as evacuants, and those that have had the most power in this respect have always been the most efficient. The hydragogue catharics, which produce the most rapid and abundant discharges from the intestins, are preferred. The object is not merely to unload the intestines, but to produce catharsis. In one case, Dr. Armstrong having taken twenty-four ounces of blood from the arm, so as to induce fainting, gave a scruple of calomel and two ounces of strong infusion of senna, containing two drachms of sulphate of magnesia, every hour till copious evacuations should be produced. "In about four hours the medicines began to operate, and several copious, dark fetid stools were discharged; from that time considerable relief was obtained, and a regular perseverance in purgatives, with mucilaginous drinks, and a small quantity of exceeding weak chicken broth, completed the cure in five days." This, certainly, is not the treatment of peritonitis. If this active practice overcame the constipation that attends this inflammation, it would have the effect, not of reducing, but of greatly increasing it; but as a means of purifying the blood, by exciting an active secretion from the intestines, the practice is intelligible.

*Emetics* were among the first remedies discovered for the treatment of this disease. In 1782, Doulcet at once checked the progress of this distemper by the free use of emetics; the hint was given him by nature herself; one of his patients, who had been just attacked, made several efforts to vomit; he gave her a large emetic, which proved salutary. He continued them, and she recovered. This practice was repeated in every case, and with such remarkable success that it was supposed Doulcet had found a specific; a second visitation, however, afterwards presented itself, and Doulcet's specific was found to be of no use. Richter, Tonnellé, and Cruveilhier, also found emetics, in some cases, very useful. This practice, then, fails in some instances and succeeds in others. Can any reason be assigned for this difference of success? Dr. Ferguson, we think, has given the true explanation. Emetics are efficient "when the violence of the malady has fallen on the liver especially, and when there is early nausea and spontaneous vomiting." "In puerperal fever I have already noticed the frequency of nausea, biliary derangement, dusky staining of the skin, and jaundice; and it must not be forgotten that Gaspard and Cruveilhier have proved that one of the modes by which infection of the blood is relieved is through the liver. On these grounds, and within these limits, the use of emetics will be found rational and beneficial."

*Diuretics* have received very little attention in the treatment of puerperal fever; yet, as evacuants, I believe them to be very efficient. In some cases I have found the free use of nitrate of potass of great value; and although as yet my experience of it as an evacuant in puerperal fever is too limited to speak with sufficient confidence, I am inclined to think that it is well worthy of a more decided notice from the profession. Its action may be explained on the same principle as emetics—the infection of the blood being relieved through the kidney in place of the liver.

*Mercury* is the only medicine that may be considered strictly as an antiphlogistic, and it is the only remedy of doubtful efficacy. Patients have been salivated with mercury, and no remission of the symptoms followed. Dr. Ferguson says—"With regard to the use of mercury in puerperal fever, I think that as a purge it may be used in all the forms with advantage, but not as a means of affecting the constitution." Dr. Collins used mercury in combination with ipecacuan in large doses, four grains of each, every second, third, or fourth hour. In other instances, he gave a scruple of calomel every second or third hour. "One patient took more than an ounce." Dr. Collins further remarks—"I could not observe any better effect from the large doses than the small; the system was not more speedily influenced; and when they did so act, it was often with violence, so as to endanger the destruction of the soft parts about the palate." In a note he observes—"It is supposed by some practitioners that when we can get the system under the influence of mercury recovery is certain. This is not the fact, as I have seen several cases, where death took place under these circumstances. It is, notwithstanding, a very favorable occurrence." Mercury, therefore, seems to be the least efficient of the remedies employed.

*Antimonials* have been also employed. Boer used an unknown preparation extensively in the Vieuna Hospital. Dr. Gooch supposed it to be "Kermes mineral;" Dr. Ferguson, "James's powder." Its action was chiefly on the skin, and was found to have a most beneficial effect. Dr. Denman derived considerable advantage from tartar emetic in the treatment of puerperal fever, acting both as an emetic and in increasing the action of the exhalants.

In the treatment, then, of puerperal fever by evacuants, the first question to decide is—Can the patient bear any evacuation? The extreme degree, the ataxic form of the fever, admits of none. In the less severe varieties we may use some one or other of them, and it only remains to determine which we shall select. If the pulse justify depletion, it is the most efficient when promptly adopted. Active saline purgatives may also be used in such cases with advantage. In more severe cases the lancet may not be employed, and yet other evacuants may be useful, which Nature herself will sometimes indicate. The fever may commence with an attack of diarrhœa, and if, in place of checking it, we rather maintain the evacuation, the patient will recover. Bilious vomiting taught Doulcet the value of emetics, and so, with regard to the efforts to throw off the disease by other channels, the symptoms will point out the treatment. There are cases, however, in which no evacuation dare be attempted,—in which stimulants alone will save the patient; and when this course is pursued it must be a bold one—a hesitating practice is perfectly useless. Dr. Copland gave from eight to sixteen grains of camphor every four or five hours. On the same principle opium may be given to any extent short of narcotism; but by far the most efficient stimulant is turpentine, because it combines in itself a double power. It supports the constitution, or rather rouses its energies against the poison, while it acts as an evacuant in removing it. Turpentine may be combined with castor-oil, in half ounce doses, and given every three or four hours with great advantage. As a local application, also, it contributes greatly to relieve the abdominal pain which the patient experiences. With regard to this medicine, I have no hesitation in stating, with Dr. Copland, that "*there is certainly no remedy so efficacious as a decided and judicious use of spirit of turpentine.*" With these observations we shall conclude the discussion of this perplexed subject, fully aware that in our attempts to unravel some of its difficulties we originate new questions for discussion and controversy. If, however, we have succeeded in rendering intelligible the principles upon which it appears to us the the most efficient plans of treatment have been successful, our object has been fully accomplished.—*Medical Gazette.*

